# Supplementary material for: Response of Extremely Small Populations to Climate Change—A Case of Trachycarpus nanus in Yunnan, China
Source: Biology (Basel). 2024 Apr 5;13(4):240. doi: 10.3390/biology13040240 (PMC11048604; doi:10.3390/biology13040240)
Supplement: Supplementary file 1 [file biology-13-00240-s001.zip › Supplementary Materials/Table S1.pdf]

Table S1. Alternation trends in the longitude, latitude, and altitude of *T. nanus* during different periods.

Period	Longitude / (°)	Latitude / (°)	Altitude / (m)
LIG	101.313013	25.442303	2541
LGM	101.491190	25.479192	2044
MH	101.381510	25.494053	2287
Current	101.354827	25.549136	2251
2030s-SSP1-2.6	101.361941	25.434287	2450
2030s-SSP2-4.5	101.454690	25.522029	2620
2030s-SSP5-8.5	101.345316	25.472278	2518
2050s-SSP1-2.6	101.410700	25.434495	2578
2050s-SSP2-4.5	101.287877	25.526486	1969
2050s-SSP5-8.5	101.317942	25.457604	2658
2070s-SSP1-2.6	101.382936	25.480248	2305
2070s-SSP2-4.5	101.276269	25.454919	2261
2070s-SSP5-8.5	101.378872	25.466853	2311
2090s-SSP1-2.6	101.328259	25.524572	2445
2090s-SSP2-4.5	101.320784	25.436330	2502
2090s-SSP5-8.5	101.514749	25.377763	1887
