# Supplementary material for: Response of Extremely Small Populations to Climate Change—A Case of Trachycarpus nanus in Yunnan, China
Source: Biology (Basel). 2024 Apr 5;13(4):240. doi: 10.3390/biology13040240 (PMC11048604; doi:10.3390/biology13040240)
Supplement: Supplementary file 1 [file biology-13-00240-s001.zip › Supplementary Materials/Table S2.pdf]

Table S2. Migration distance of the centroid in the past and under different scenarios in the future.

Type	Period	Migration distance / Km
Past	LIG–LGM	17.71
	LGM–MH	10.72
	MH–Current	6.86
SSP1-2.6	Current–2030s	13.28
	2030s–2050s	4.71
	2050s–2070s	5.92
	2070s–2090s	7.35
SSP2-4.5	Current–2030s	10.13
	2030s–2050s	16.10
	2050s–2070s	8.34
	2070s–2090s	4.80
SSP5-8.5	Current–2030s	8.92
	2030s–2050s	3.14
	2050s–2070s	5.98
	2070s–2090s	16.67
