# Supplementary figures and images for: Response of Extremely Small Populations to Climate Change—A Case of Trachycarpus nanus in Yunnan, China
Source: Biology (Basel). 2024 Apr 5;13(4):240. doi: 10.3390/biology13040240 (PMC11048604; doi:10.3390/biology13040240)

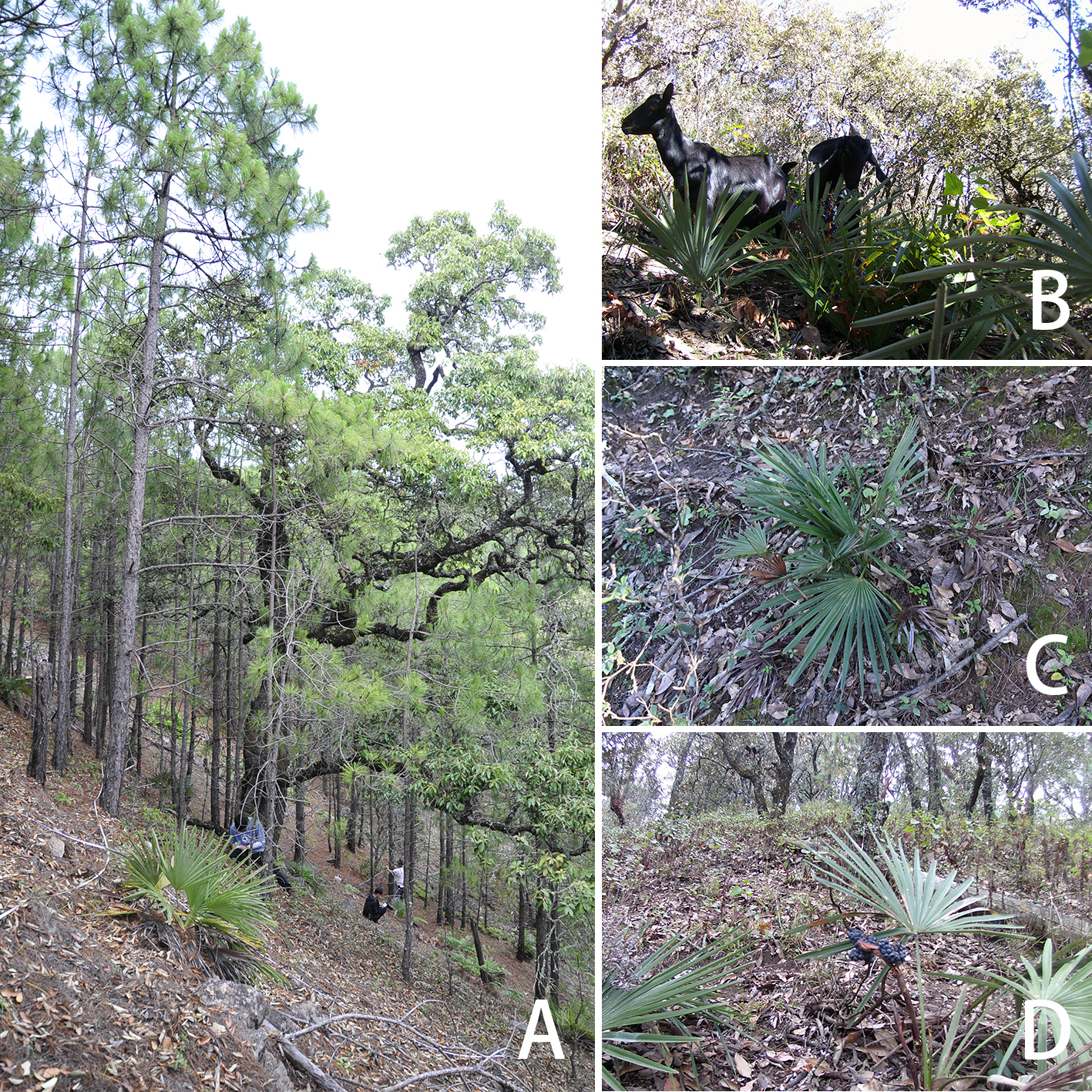

Supplement: Supplementary file 1 [file biology-13-00240-s001.zip › Supplementary Materials/Figure S1.tif]

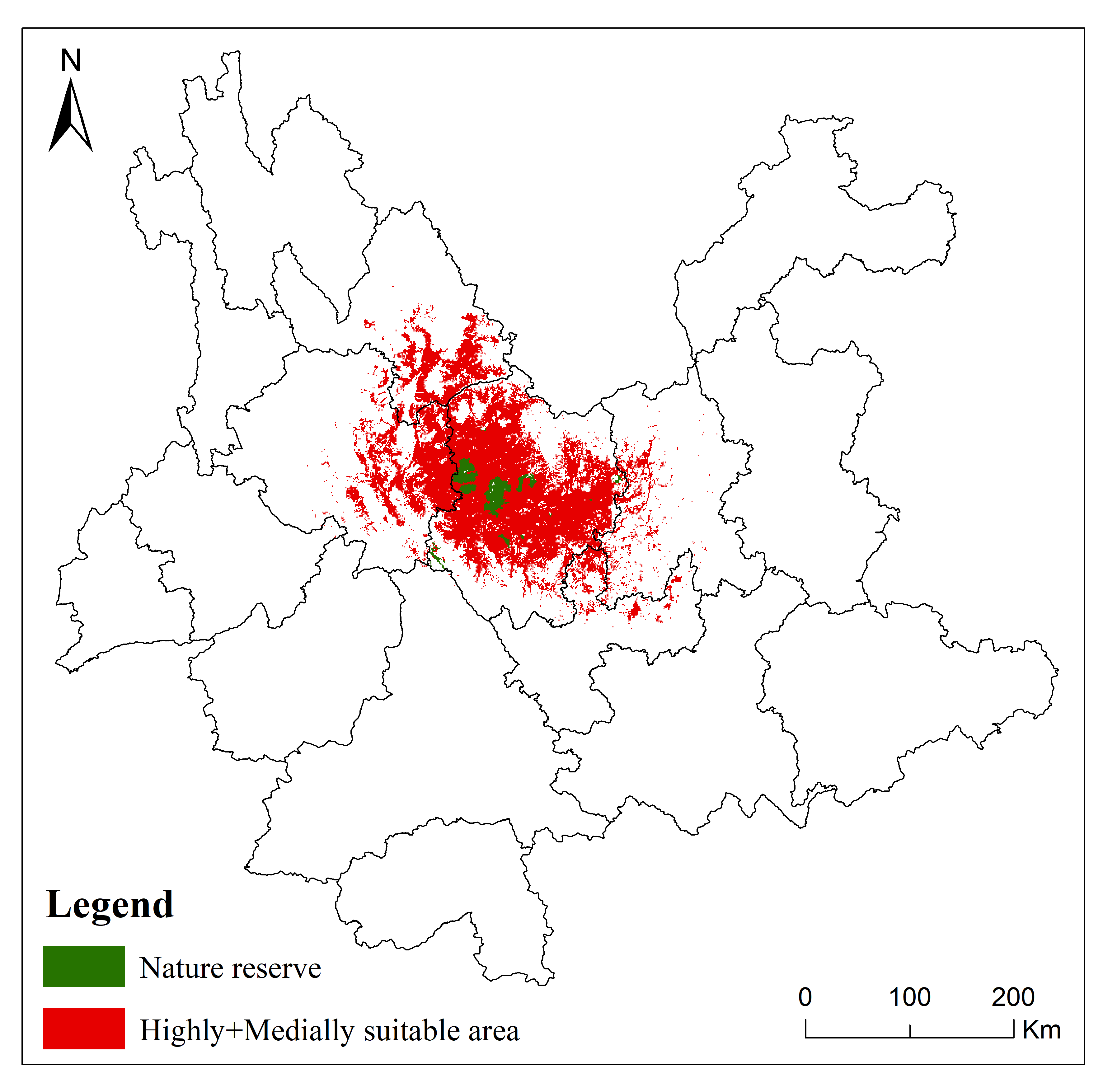

Supplement: Supplementary file 1 [file biology-13-00240-s001.zip › Supplementary Materials/Figure S3.tif]
